# Boxing Punch Detection and Classification Using Motion Tape and Machine Learning

**DOI:** 10.3390/s25165027

**Published:** 2025-08-13

**Authors:** Shih-Chao Huang, Taylor Pierce, Yun-An Lin, Kenneth J. Loh

**Affiliations:** 1 Active, Responsive, Multifunctional, and Ordered-Materials Research (ARMOR) Laboratory, Department of Structural Engineering, University of California San Diego, La Jolla, CA 92093, USA; shh027@ucsd.edu (S.-C.H.); yul095@ucsd.edu (Y.-A.L.); 2 Department of Electrical and Computer Engineering, University of California San Diego, La Jolla, CA 92093, USA; tpierce@ucsd.edu

**Keywords:** classification, InceptionTime, MiniRocket, movement, sports, supervised learning, time series transformer, training, wearable sensors

## Abstract

The objective of this study is to classify the types of boxing punches using machine learning algorithms that processed skin-strain time history measurements from a self-adhesive, elastic fabric, wearable sensor called Motion Tape. A human participant study was designed to capture movements during boxing training. Subjects were asked to perform multiple sets of punches during the entire test, which consisted of jabs and hooks with and without striking a heavy bag. The collected Motion Tape data was used to train and compare time series classification algorithms to identify the types of punches performed and associated conditions. The results demonstrated that Motion Tape, in combination with machine learning techniques, could effectively classify different punch types based on skin-strain measurements. These findings highlighted the potential of the system as an effective tool for human performance analysis in sports and biomechanics applications.

## 1. Introduction

Boxing is a centuries-old sport and continues to remain popular across different countries [1]. Working with a personal coach and receiving immediate feedback has been the predominant technique for one to learn how to box or to improve their skills. An alternative is to leverage sports video analysis, which is often used post-match to learn from previous mistakes and successes. In either case, the feedback from coaches is subjective. Quantitative metrics (e.g., the number of punches thrown) during training or a match cannot be determined unless the coach manually records them, which may be susceptible to human error and takes away their attention from providing skills feedback.

More recently, boxing training has evolved to not require the physical presence of coaches. For example, PunchLab lets users strap their smartphone on a heavy bag, and coaches can provide remotely delivered visual and verbal instructions during training [2]. It is possible to acquire some punching metrics (e.g., types, speed, and counts of punches, among others) during tele-boxing training, which is helpful for assessing overall boxing performance. Other more sophisticated approaches adopt either vision-based or sensor-based approaches to analyze boxing performance. First, vision-based approaches include, for example, the use of overhead low-resolution depth images to classify six basic boxing punches (i.e., straight, hook, and uppercut for both the rear and lead hands) [3]. The method extracted transition invariant spatial-temporal features from the relative position of the boxer’s upper body parts and classified boxing punch types with 96% accuracy using a hierarchical coarse-to-fine support vector machine (SVM) classifier [4]. Furthermore, Stefański et al. [5] classified three classes of boxing punches in Olympic boxing using RGB (red, green, and blue) cameras and computer vision algorithms. After applying data augmentation techniques to the dataset, the best *F*_1_ score (i.e., a measure of the model’s accuracy of a certain class on a dataset considering both precision and recall) achieved for one of the three classes was 0.94 [5]. Although these techniques achieved high accuracy, a limitation is that cameras may not be available or positioned appropriately for every training environment or match.

Second, in lieu of cameras, the alternative approach of using small and portable sensors means that they can be potentially used in almost every training environment. Sensors are often placed on either the heavy bag or directly on boxers. For example, Vales-Alonso et al. [6] proposed an unsupervised learning approach to perform punch detection, clustering, and evaluation of a boxer’s unpredictability by analyzing data from accelerometers installed inside the heavy bag. Their framework achieved ~ 90% accuracy for punch clustering, and real-time results could be computed and shown during each training session [6]. Buśko et al. [7] measured punching force, punch location on the bag, and reaction time with accelerometers and gyroscopes embedded in the heavy bag. The relative error of force measurements achieved was 3% [7]. However, a limitation of placing the sensors on the heavy bag is that these methods cannot be used in training scenarios that do not employ the heavy bag, such as during shadowboxing and sparring.

Wearable sensors with accelerometers and gyroscopes have been developed to make boxing performance tracking more broadly accessible; however, they usually only track the motion of specific body parts while ignoring the rest of the body [8]. These sensors are most frequently placed on the forearm and wrist [9]. For example, StrikeTec placed two sensors on the wrist to evaluate relevant metrics of boxing (e.g., speed, power, and punch type) [10]. However, placing sensors only on limited body parts ignores how one’s entire biomechanics could potentially affect punch delivery. Although Hanada et al. [11] placed inertial measurement units (IMUs) on the wrist and upper back for punch detection and classification. IMUs are susceptible to motion artifacts and could compromise comfort and adversely affect their movements. Therefore, a technology that can track boxers’ different body movements, assess their performance, and be comfortable to wear is still needed.

The objective of this study was to validate that the unique data streams from a low-profile, self-adhesive, elastic fabric, wearable skin-strain sensor, called Motion Tape, could be analyzed using machine learning methods to automatically classify the types of punches thrown during simulated training activities [12]. Previous work already showed that unique skin-strain measurements associated with different movements and signals correlated with the degree of muscle engagement could be acquired with Motion Tape, all without sacrificing comfort [13,14]. By leveraging Motion Tape and a custom, wireless data acquisition (DAQ) unit, a human participant study was designed to capture movements during boxing. In particular, subjects were asked to perform a total of 480 punches during the entire test, including jabs and lead hooks under different conditions (e.g., striking a target or missing). These datasets were then used for punch classification. Time series classification algorithms were compared and adopted to train machine learning models that identified punches under different conditions.

## 2. Materials and Methods

### 2.1. Experimental Details

#### 2.1.1. Materials

The materials used to fabricate Motion Tape consisted of graphene nanosheets (GNSs), which were synthesized using a water-assisted liquid phase exfoliation technique [15]), ethyl cellulose (EC, from Sigma-Aldrich, St. Louis, MO, USA), 200 proof ethyl alcohol (ETOH) (from Fisher Scientific, Pittsburgh, PA, USA), and kinesiology tape (K-Tape, from Rock Tape^TM^, Durham, NC, USA).

#### 2.1.2. Sensor Fabrication

Motion Tape fabrication was described in detail by Lin et al. [12] but is summarized here for completeness. In short, the piezoresistive nanocomposite that is at the core of Motion Tape was made by dispersing GNS in an EC solution in ETOH. An airbrush (from Paasche, Kenosha, WI, USA) was used to deposit the GNS-EC dispersion onto rectangular masked portions of commercially available K-Tape substrates to form the sensing element. Flexible conductive silver electrodes were painted at opposite ends of the nanocomposite and dried before multi-strand wires were soldered for ease of measurements [16].

#### 2.1.3. Wireless Sensing Node

**Figure 1:**
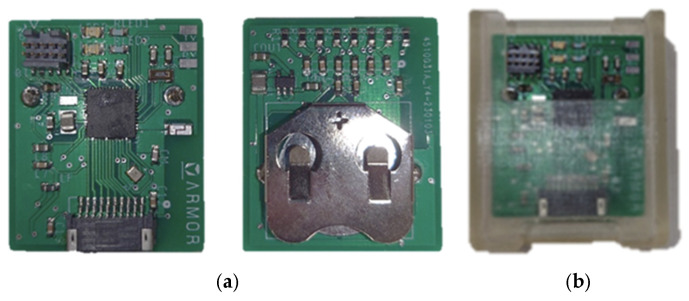
(**a**) Custom-made PCB design with (**b**) a 3D-printed enclosure for extra protection.

A customized, wireless, portable data acquisition sensing node was designed and used to record all Motion Tapes data streams during human participant studies [17]. The wireless sensing node employed a Texas Instruments CC1350 microcontroller unit (mcu) at the heart of its computational core. This mcu integrates essential DAQ functions on-chip, including a 12-bit analog-to-digital converter (ADC) with up to 8-pin multiplexing and a 2.4 GHz wireless transceiver for Bluetooth low energy (BLE) communications. The wireless sensing node also features a CR2032 coin cell battery retainer, power regulation circuitry, and a chip antenna. Voltage measurements were captured using the onboard ADC rated at a sampling frequency of ~80 Hz per channel. Up to eight sensing channels could be recorded simultaneously. These sensing streams were transmitted via BLE communication to another CC1350 board, which was connected to a laptop for data logging. Timestamps of all channels were synced with the laptop for further data processing. Figure 1 shows the custom-made printed circuit board (PCB) with a resin enclosure for providing physical protection to the electronics. The enclosure was designed and printed using a Formlabs (Somerville, MA, USA) Form 3 stereolithography (SLA) 3D printer.

#### 2.1.4. Optical Motion Capture

**Figure 2:**
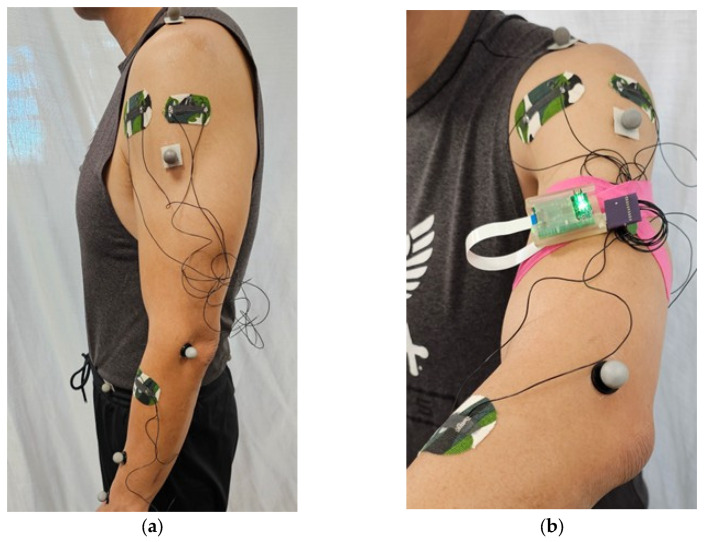
(**a**) Motion Tapes were affixed to subjects’ anterior deltoid, middle deltoid, and forearm to record relevant data streams (alongside motion capture retroreflective markers), while a (**b**) custom-made DAQ collected and wirelessly transmitted all the Motion Tape sensing streams.

Optical motion capture (mocap) was employed as a reference measurement system for quantifying the kinematic movements of subjects during testing. The mocap system consists of infrared cameras that detect retroreflective markers by emitting infrared light and capturing the reflected signal. The retroreflective markers bounce the infrared (IR) light directly back toward the camera lens, which allows the mocap software to independently track each marker’s changing positions. Therefore, retroreflective markers were affixed onto subjects (Figure 2) so that mocap could track the subject’s movements in 3D space during each trial. The full-body Plug-in Gait marker placements are based on the Newington–Helen Hayes gait model [18].

The mocap system utilized in this study is from Vicon (Yarnton, Oxfordshire, UK). The version of the Vicon software is 2.12.1. This 12-camera motion capture system captured subjects’ motion at a sampling frequency of 120 Hz and could further sample at up to 330 Hz. Since mocap is only leveraged as a reference system, the maximum sampling rate was not used. For future studies that require athletes’ biomechanical data, the maximum sampling rate can be selected to ensure higher-resolution data. Each of the Vicon Vero v2.2 cameras captures optical data with a resolution of 2048 × 1088 pixels and 3.6 ms of camera latency. Furthermore, each optical camera was equipped with an adjustable standard and a wide lens for a flexible field of view.

#### 2.1.5. Human Participant Study for Boxing

This human subject study was approved by the University of California San Diego Institutional Review Boards, Human Research Protection Program, under Project No. 191806X. Informed written consent was obtained from all participants. First, each subject wore three Motion Tapes, each affixed onto the anterior deltoid, middle deltoid, and forearm, as shown in Figure 2. All three Motion Tapes were connected to the same wireless sensing node for data collection. Second, the subject also wore a full-body set of retroreflective markers so that mocap kinematic measurements could be acquired during testing. Time synchronization of both the Motion Tape and mocap data streams was achieved using the Vicon Lock Lab analog interface. The Motion Tape data streams from the wireless sensing node on the human subject were wirelessly transmitted to a wireless receiver connected to the Vicon Lock Lab.

The human participant study was designed so that subjects performed multiple sets of different types of punches, and the entire set of different conditions are listed in Table 1, including (1) normal jabs; (2) jabs while holding 5 lb dumbbells; (3) jabs that struck a body opponent bag (BOB); (4) normal lead hooks; (5) lead hooks while holding 5 lb dumbbells; and (6) lead hooks that struck the BOB. Subjects performed 20 repeated punches of each type to form a trial set. In total, 480 punches were performed during the entire test, where different numbers of trials were performed for the various punches.

### 2.2. Data Processing Method

To construct the dataset for punch type classification, Motion Tape-normalized electrical resistance measurements (*ΔR_n_*) were obtained as follows:

*ΔR*_*n*_ = (*R*_*i*_ − *R*_0_)/*R*_0_ (1)

where *R_i_* is the resistance of Motion Tapes at each time instance *i*, and *R*_0_ is the baseline resistance of Motion Tape. In this study, *R*_0_ is the recorded resistance when subjects remained still in a neutral position before performing punching in each trial. The time history record of each trial set was segmented using a punch detection algorithm proposed by Vales-Alonso et al. [6], where each punch was then considered as a single sample in the dataset. The punch detection algorithm will be introduced and explained in detail in Section 2.3. In short, an adaptive threshold was calculated using the baseline resistance measurements. Once resistance measurement exceeded this adaptive threshold, the Motion Tape time history portion of each punch was then detected and segmented. Appropriate parameters were tuned to enable the punch detection algorithm to detect as many punches as possible. Instead of segmenting the time history data of each punch manually, this detection algorithm was adopted because the entire data post-processing procedure could be achieved without human intervention, which is desirable so that real-time assessment and feedback can be provided to boxers during their training.

Next, each punch time history was resampled to have equal sequence length, because many time series classification models require all input data to have the same dimension and sequence length. Among all time-series classification models investigated in this study, the Time Series Transformer (TST) was the only model that did not require input data of equal dimension and sequence length because of the base model architecture and the padding mask used in the aforementioned model [19]. More details of TST will be discussed in Section 2.4. Eventually, a total of 450 samples were segmented to construct the dataset, where there were 303, 82, and 65 samples in the training, validation, and testing sets, respectively.

### 2.3. Punch Detection Method

Being capable of detecting and counting the number of punches is the foundation of constructing a performance assessment system for boxing. Boxers rely on the number of punches performed, either counted manually or provided by commercial boxing punch trackers, to develop and adjust their training (and fight) plan. On top of that, punch detection enables automatic segmentation of data streams of punches, which prevents the whole performance assessment system from taking irrelevant data streams as inputs.

In this study, a punch detection algorithm, proposed by Vales-Alonso et al. [6] and shown in Algorithm 1, was employed for punch detection and segmentation as the first step during data processing. The punch detection algorithm was originally applied to accelerometer data, but it was adapted with some parameter tuning for processing Motion Tape data. The algorithm works by detecting a punch when the incoming data passes a certain threshold, which is calculated adaptively (to account for noise) using Motion Tape baseline measurements (or also referred to as calibration data in Vales-Alonso et al. [6]). The power of Motion Tape data streams was calculated as follows:

(2) $$p_{i}=\frac{1}{S}\sum_{s=1}^{S}{\left\|{{∆R}_{n}}_{i}^{\left(s\right)}\right\|}_{2}$$

where S is the total number of sensors, and ${{∆R}_{n}}_{i}^{\left(s\right)}$ is the normalized resistance of the *s*th sensor at instance *i* calculated using (1). Although $p_{i}$ may be directly used for punch detection, it tends to perform poorly if the sensor data is noisy. Therefore, the Mahalanobis distance was introduced to supplement the punch detection procedure [20]. A multivariate Gaussian model was fit to baseline Motion Tape data, where the mean $\mu_{C}$ and covariance matrix $\Sigma_{C}$ could be calculated using the following equation:

(3) $$\mu_{C}=\frac{1}{L}\sum_{i=1}^{L}x_{i}$$

(4) $$\Sigma_{C}=\frac{1}{L-1}\sum_{i=1}^{L}\left(x_{i}-\mu_{C}\right){\left(x_{i}-\mu_{C}\right)}^{T}$$

where L is the number of samples of Motion Tape data in the baseline trial, and $x_{i}$ is the normalized resistance of Motion Tapes at instance *i*. The Mahalanobis distance of a new point x to the cluster center $\mu_{C}$ was then:

(5) $$d\left(x_{i},\;\mu_{C}\right)={\left[{\left(x_{i}-\mu_{C}\right)}^{T}{\Sigma_{C}}^{-1}\left(x_{i}-\mu_{C}\right)\right]}^{\frac{1}{6}}$$

It is noted that the exponent of the original Mahalanobis distance equation is 1/2 instead of 1/6. The exponent of (5) was modified here for scaling the magnitude of the Mahalanobis distance. The appropriate value of the exponent may be different for different sensors and different datasets, and thus should be carefully tuned. Among all $x_{i}$ in the baseline Motion Tape data, the maximum Mahalanobis distance is defined as follows:

(6) $$d_{C}=\underset{i=1,\;\cdots ,L}{\max}d\left(x_{i},\;\mu_{C}\right)$$

After all the necessary inputs were calculated, punch detection could be performed with Algorithm 1 [6]. In Algorithm 1, *S* is the number of Motion Tapes, and *M* is the minimum number of consecutive time steps that the Mahalanobis distance needs to be larger than the threshold when a punch section is confirmed. In addition, *γ* enables the punch detection algorithm to search for the end of the punch section dynamically in a longer time frame, because the duration of each punch section is slightly different. The other parameters of *ε*_min_, *ε*_max_, and Δ*_ε_* are used to define the threshold limit in the punch detection algorithm. It should be noted that the parameters used in this work were selected to optimize punch detection results (i.e., punches were detected as many as possible), where *S*, *M*, *ε*_min_, *ε*_max_, *γ*, and Δ*_ε_* were selected to be 3, 8, 9, 11.5, 1.5, and 0.001, respectively.
**Algorithm 1. Punch detection procedure by Vales-Alonso et al. [6]****1****Input**: Points $\left\{x_{i}\right\}$, Powers $\left\{p_{i}\right\}$, Distances $\left\{d_{i}\right\}$, Calibration cluster $\{\mu_{C}$, $\Sigma_{C}$, $d_{C}\}$**2****Output**: Punch times $\{t_{1}$, $t_{2}$, ⋯, $t_{N}\}$**3****Parameters**: S, M, $\varepsilon_{min}$, $\varepsilon_{max}$, γ, $\Delta_{\varepsilon }$

**4**$d_{min}=\left(1+\varepsilon_{min}\right)d_{C}$;**5**$\varepsilon \leftarrow \varepsilon_{max}$;**6**${th}_{i}\leftarrow d_{min}$ for all *i*;**7****repeat****8****for** *all i* **do**
//Possible punch section**9**$v_{i}\leftarrow 1$ if $d_{i}\geq {th}_{i}$, otherwise 0;**10****end**
//Punch confirmation**11**$v_{i}\leftarrow 0$ if less than M consecutive 1s;**12****for** *each i such that* $v_{i}=1\;and\;v_{i-1}=0$ **do**
//Threshold updating**13**${th}_{i+k}\leftarrow max\left\{{th}_{i+k},\;\varepsilon d_{C}\right\}$ for k=0 to $\left\lfloor \gamma M\right\rceil$;**14****end****15**$\varepsilon \leftarrow \varepsilon -\Delta_{\varepsilon }$;**16****until** $\varepsilon \geq \varepsilon_{min}$;


//Time extraction**17**t←ϕ;**18****for** *each i such that* $v_{i}=1\;and\;v_{i-1}=0$ **do****19**l← number of consecutive 1s starting at index *i*;**20**Append time of index ${argmax}_{i\leq k<i+l}\left\{p_{k}{d_{k}}^{2}\right\}$ to t;**21****end**

**22****Return** t

### 2.4. Punch Classification Models

After the datasets were acquired and processed, several machine learning models were trained to classify the types of punches. Machine learning (ML) methods with automatic feature selection and extraction are particularly appropriate for this study, because useful features for multivariate time series data can sometimes be hard to find. Abundant domain knowledge and a vast amount of time may be required to obtain suitable features for multivariate time series classification problems. Therefore, ML-based methods with automatic feature selection were preferred and selected in this study to facilitate the model development process and to achieve better overall model performance.

There was a total of three ML algorithms adopted in this study to showcase that Motion Tape data streams from different muscle groups could be used to identify different types of punches with high accuracy and thus serve as suitable features for punch type classification. Time Series Transformer (TST), **MINI**mally **R**and**O**m **C**onvolutional **KE**rnel **T**ransform (MiniRocket), and InceptionTime are all state-of-the-art ML models for time series classification based on performance on public benchmarks [19,21,22,23,24]. It should be noted that these ML models were all implemented by using the Python package called *tsai*, which is an open-source ML library with a variety of techniques for time series tasks, including regression, classification, and forecasting [25]. Furthermore, the hyperparameters of all ML models were fine-tuned and optimized using the Python package called *Nevergrad* to achieve the best possible model performance given the available time and computational budget [26].

First, *Nevergrad* is a gradient-free platform suitable for solving black-box optimization problems developed by the Meta research group [26]. In particular, *Nevergrad* can complete hyperparameter fine-tuning without wasting computational resources for sets of hyperparameters that are less likely to produce better model performance, which makes it more effective than other less systemic or efficient methods (e.g., trial and error or grid search). It also contains a large library with a plethora of optimization algorithms for both continuous and discrete variables, such as the Covariance Matrix Adaptation evolution strategies (CMA-ES), Bayesian optimization, particle swarm optimization (PSO), constrained optimization by linear approximation (Cobyla), and fast genetic algorithms (Fast-GA), among others [27,28,29,30,31]. *Nevergrad* leverages its library by employing an automatic algorithm selection pipeline so that appropriate optimization algorithms will be adopted according to different criteria, including the types of variables, optimization results in the first few runs, and available computational budget, among others.

The final hyperparameters that are optimized using *Nevergrad* and used to train each machine learning model used in this study are summarized in Table 2. The hyperparameters not shown in Table 2 are default values set in the *tsai* package and can be found in [25]. It is noted that the hyperparameter *Conv ks* in Table 2 was used to control the kernel sizes of different convolutions in the InceptionTime model and not simply the kernel size of a single one-dimensional convolution.

#### 2.4.1. Time Series Transformer

TST is a deep learning transformer-based model inspired by the work of Vaswani et al. [32], which is the foundation of the very successful large language models (LLMs, such as GPT-4 [33]) in the natural language processing (NLP) field. Instead of employing the decoder [32], Zerveas et al. [19] only kept the encoder and substituted the decoder with different output layers according to the types of downstream problems (i.e., regression or classification problem). The reason behind this modification is that the decoder is usually used to generate outputs for generative tasks, such as translation and question answering in NLP, which is not required for either regression or classification problems.

**Figure 3:**
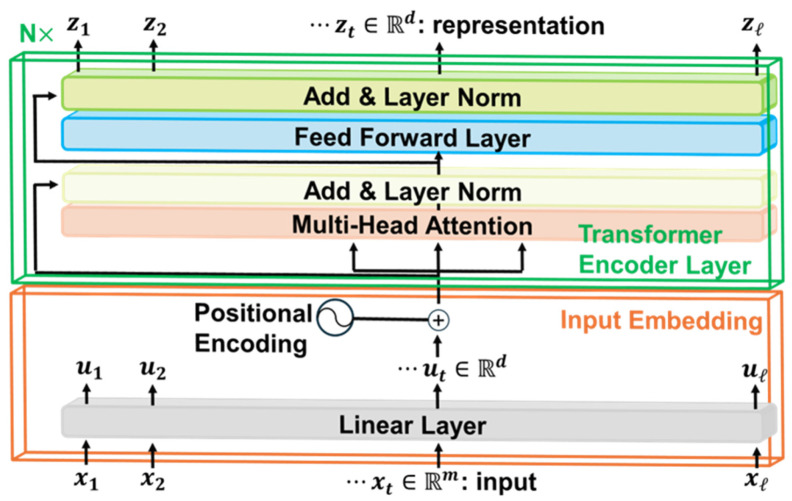
The generic model architecture of TST is shown. Input multivariate time series data $x_{t}$ is linearly projected to create an input embedding $u_{t}$ and then fed to transformer encoder layers to output final representation $z_{t}$.

The generic model architecture of TST is shown in Figure 3. The input multivariate time series data $X\in R^{l\times m}$ consists of a sequence of $x_{t}\in R^{m}$ for each time step, where l is the total length of the time series data, and m is the dimensionality of the input data. It is noted that TST is the only ML model in this study that does not require X to be resampled so that all time series data in the entire dataset have the same time length and interval between time steps. Instead, variables in each $x_{t}$ after the total time length l are padded with zeros. This is performed with padding masks that indicate which parts of the time series data should be ignored by the TST, which add large negative values to generate low attention scores for the padded position, so that the model can deal with time series data of varying lengths. Afterwards, $x_{t}$ is standardized by each variable and linearly projected to $u_{t}$ with a dimensionality of d, which can be described as follows:

(7) $$u_{t}=W_{p}x_{t}+b_{p}$$

where both $W_{p}\in R^{d\times m}$ and $b_{p}\in R^{d}$ are learnable parameters that will be trained during the model training process. $u_{t}$ is then added by the positional encoding with learnable parameters and becomes the input embedding, which serves as the input to the subsequent encoder layers. As for the encoder, all elements and operations follow the original algorithm [32]. Finally, the final representation $z_{t}$ is fed to a fully connected layer, followed by a *softmax* layer to predict classes for the classification problem.

#### 2.4.2. MiniRocket

MiniRocket is the only non-deep learning method used in this study [21]. MiniRocket essentially transforms the input data into useful features. More specifically, a small and fixed set of convolutional kernels with different weights is applied to the input data to produce the feature map. In addition, the feature map is passed through a proportion of positive values (PPVs) pooling to obtain the transformed features, which can be described as follows:

(8) $$PPV\left(X*W\right)=\frac{1}{n}\sum \left[X*W>b\right]$$

where ∗ denotes convolution, W is kernel, b is bias, n is the number of elements in the convolution output X∗W, and $\left[\cdot \right]$ denotes the indicator function. According to Dempster et al. [21], bias b is drawn from the quantiles of the convolution output. The transformed features are then used to train another classifier for classification problems.

For example, a fully connected layer was trained using the transformed features for the final classification results in this study. Although Dempster et al. [21] used ridge regression and a logistic regression classifier, utilizing a fully connected neural network (as implemented in the *tsai* package), should achieve overall better classification results and provide more flexibility in terms of fine-tuning the complexity of the classifier.

#### 2.4.3. InceptionTime

InceptionTime is a convolutional neural network (CNN)-based deep learning method proposed by Fawaz et al. [22]. InceptionTime is an ensemble consisting of five deep learning models with the same architecture design but with different weight values randomly initialized, which helps mitigate high standard deviations in accuracy observed when only one model instance is trained [22].

For each individual model in InceptionTime, several Inception modules, first proposed by Szegedy et al. [34], can be stacked to extract time-invariant features for time series classification. Each of the Inception modules converts the input multivariate time series data to a low-dimensional representation using a 1D convolution filter within the bottleneck layer. Convolutions with varying filter sizes are then used to form the multivariate time series output. The design of the Inception module enables InceptionTime to not only reduce dimensionality with a bottleneck layer so that longer filters can be applied with a smaller number of parameters to be learned, but also provides more flexibility as compared to traditional CNN models due to convolutions of varying filter sizes.

**Figure 4:**
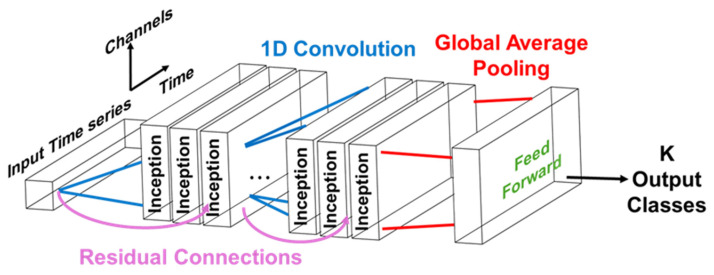
The model architecture of InceptionTime is shown. The input multivariate time series data passes through Inception modules, and global average pooling then outputs the feature map for final classification.

**Figure 5:**
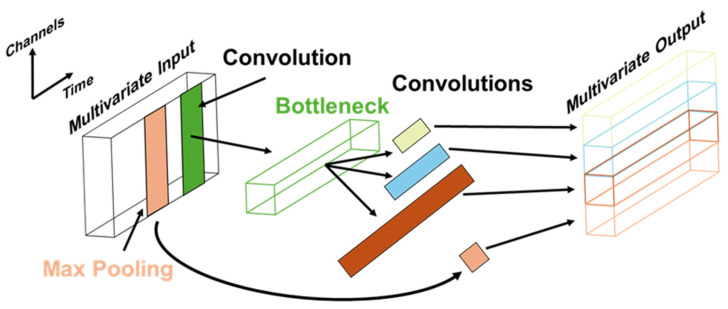
The components of the Inception modules are shown. Input multivariate time series data is first fed to the bottleneck layer for dimensionality reduction and then concatenated after convolution operations.

In addition, residual connection is applied to mitigate the vanishing gradient problem by adding the output of the previous Inception module directly to another later Inception module [35]. Subsequently, global average pooling is applied to convert the size of the output of Inception modules so that the feature map can then be passed to the final fully connected layer for predicting output classes [36]. The InceptionTime and Inception module architectures are illustrated in Figure 4 and Figure 5, respectively.

## 3. Results and Discussion

### 3.1. Dataset Visualization

**Figure 6:**
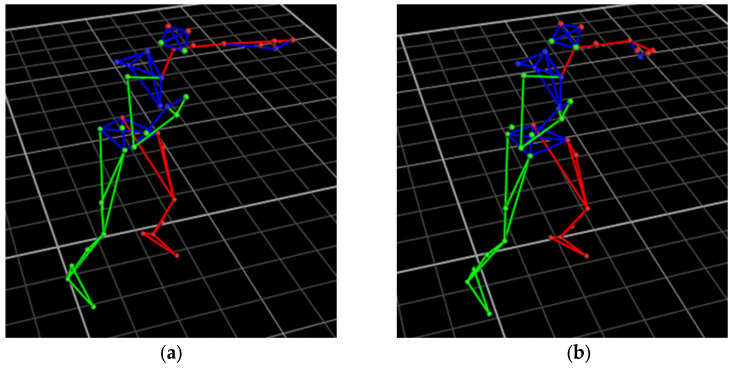
Subjects were asked to perform (**a**) jabs and (**b**) lead hooks under different conditions in each trial, as shown by the mocap recordings. The human biomechanical model is represented as simplified stick figures, where green, blue, and red correspond to the right, center, and left of subject’s trunk, respectively.

**Figure 7:**
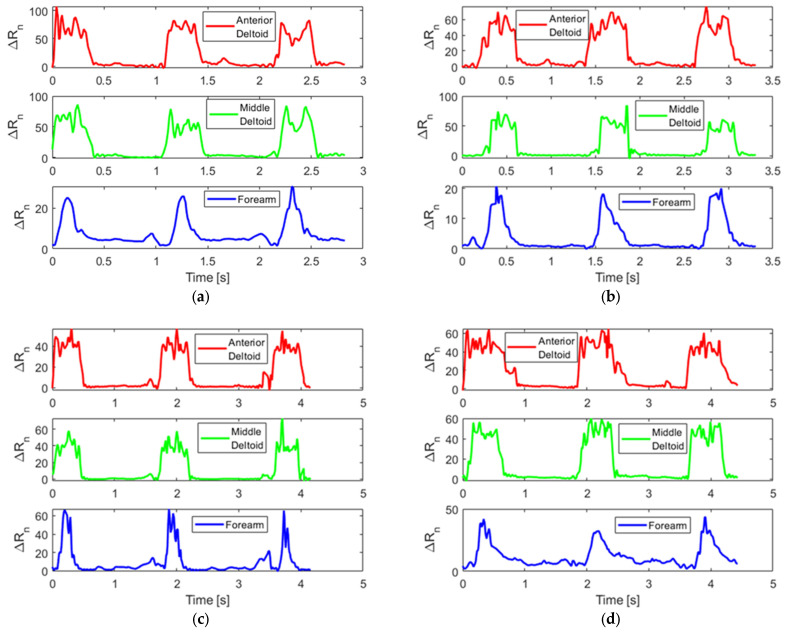

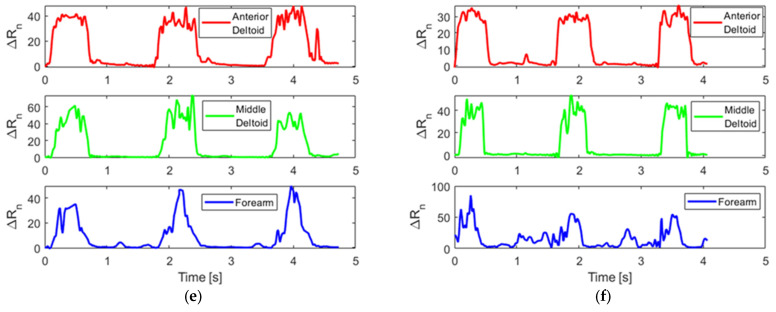
Exemplary Motion Tape time histories of three consecutive punches of (**a**) jabs, (**b**) jabs while holding 5 lb dumbbells, (**c**) jabs striking BOB, (**d**) lead hooks, (**e**) lead hooks while holding 5 lb dumbbells, and (**f**) lead hooks striking BOB.

As mentioned in Section 2.1.5, subjects wore Motion Tapes over different muscle groups during the human participant study and performed different types of punches and conditions as shown in Figure 6 and summarized in Table 1. Figure 7 shows representative Motion Tape normalized resistance time histories corresponding to the different punches thrown. The time history data streams shown in Figure 7 were randomly selected. The sensor measurements for each punch type may fluctuate slightly because there could be some variance when the subject threw a punch each time. However, the waveforms of the sensor measurements are similar for the same type of punch. It can be observed from Figure 7 that punches in this multivariate time series dataset are hard to differentiate just by visual inspection, because some of the time series data waveforms were quite similar to one another. It could be possible that some basic statistics (e.g., maximum and mean) of each punch can be used to classify punches; however, this criterion may only work for the training dataset and will fail when basic statistics drift while Motion Tape data waveforms remain similar. For example, the maximum sensor measurements may be different for different subjects, because one’s muscle strength could be higher than another individual’s; therefore, such a predefined criterion for another person would not be robust because of data drift. On the other hand, achieving pattern recognition using ML methods is superior, not only because boxing punch type classification is still possible with data drift but also because the ML methods employed in this study are able to perform automatic feature selection and can consider more complex features across different dimensions.

### 3.2. Punch Detection

The effectiveness of the chosen punch detection algorithm on the Motion Tape dataset was reported by calculating punch detection accuracy ${Acc}_{punch\_k}$, recall $R_{k}^{D}$, precision $P_{k}^{D}$, and *F*_1_ score $F_{1\_k}^{D}$ of each class, which could be evaluated using the following metrics:

(9) $${Acc}_{punch_{k}}=\;\frac{D_{k}}{D_{k}+U_{k}+N_{k}}$$

(10) $$R_{k}^{D}=\frac{D_{k}}{D_{k}+U_{k}}$$

(11) $$P_{k}^{D}=\frac{D_{k}}{D_{k}+N_{k}}$$

(12) $$F_{1_{k}}^{D}=\frac{2R^{D}P^{D}}{R^{D}+P^{D}}$$

where $D_{k}$ denotes the number of detected punches, $U_{k}$ denotes the number of undetected punches, and $N_{k}$ denotes the number of events misclassified as an actual punch for each class k.

The evaluation metrics for the chosen punch detection algorithm were calculated and summarized in Table 3. Table 3 shows that the punch detection algorithm was capable of detecting punches with high success rates, even though the sensing streams contained noise (Figure 7). The overall accuracy and *F*_1_ score were found to be 90.5% and 95%, respectively. Furthermore, the difference between the overall recall and precision was small, which indicates that there was no significant imbalance regarding undetected punches and events misrecognized as actual punches. The chosen punch detection algorithm was able to rule out both false positive and false negative cases with high success rates. Although the punch detection results for lead hooks striking BOB had lower accuracy and showed higher chances of misrecognizing an event as an actual punch, this issue could potentially be resolved in the future by finetuning the punch detection algorithm parameters or by exploring other algorithms.

### 3.3. Punch Classification

**Figure 8:**
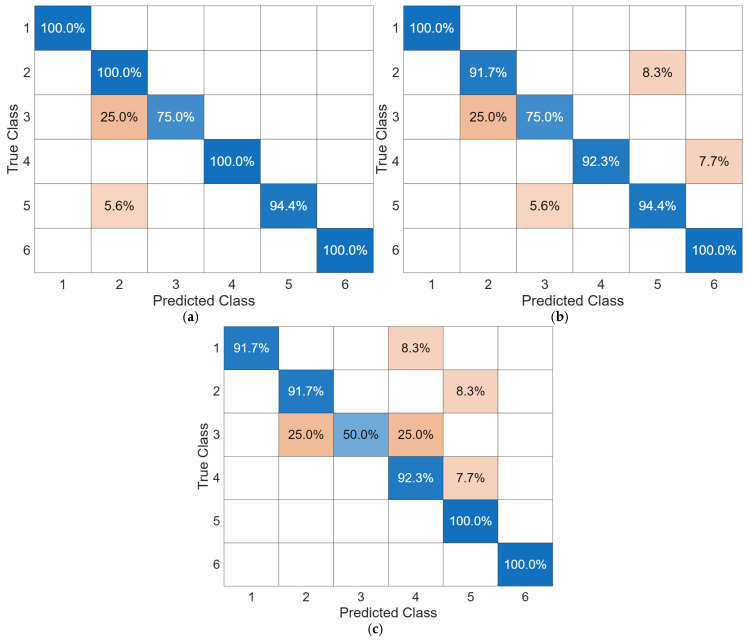
Confusion matrices of (**a**) TST, (**b**) MiniRocket, (**c**) InceptionTime. For the numeric labels in each confusion matrix, the True and Predicted classes refer to the testing Sets 1 to 6, which are “Jabs”, “Jabs (5 lb)”, “Jabs (BOB)”, “Lead hooks”, “Lead hooks (5 lb)”, and “Lead hooks (BOB)”, respectively. A colormap was assigned to different values in the confusion matrices for data visualization purposes.

After punches were detected, the results were fed into the three ML algorithms to perform punch type classification. The accuracy results are summarized in Table 4. The confusion matrices of all the models are shown in Figure 8. It can be observed that TST outperforms other algorithms and achieves an overall accuracy of 96.9%. It can also be observed that the classification accuracies for all three ML algorithms were lower for the case of jabs striking BOB, which implies that the trained model may not generalize well across different contact scenarios or environmental setups. This issue is expected to be mitigated by constructing a larger and more diverse dataset under different testing scenarios, which will be explored in a future study. Furthermore, misclassifications can also be observed using the confusion matrices shown in Figure 8. For example, jabs while holding 5 lb dumbbells tend to be misclassified as lead hooks while holding 5 lb dumbbells. This problem could potentially be solved by making sensor measurements of jabs and lead hooks more distinguishable (i.e., by adjusting the location of Motion Tapes or wearing more sensors). Therefore, these classification results showed that TST was capable of classifying boxing punches by employing Motion Tape data streams and has the potential to be used in a real training scenario.

### 3.4. Discussion of Limitations

The current study investigated the potential of utilizing Motion Tape as an alternative wearable sensing platform for athletes, and improvements of certain system components are needed before this technology can be used broadly and also across different sports and athletic activities. First, the limited amount of data and testing scenarios considered could lead to model overfitting and concern that the model does not generalize well across different users or testing scenarios. Therefore, a large-scale study involving more users and testing scenarios is planned in the future to ensure performance consistency. Second, although this study demonstrated that Motion Tape could be an alternative wearable sensor for athletic training, the soldering joints on Motion Tapes (i.e., at the electrode connecting the multi-strand wire to the nanocomposite sensing element) could break during intense, dynamic movements. To address this issue, a wire-free version of Motion Tape is currently under development. Third, an ablation study has yet to be conducted to assess the contribution of each system component, such as the impact of Motion Tape signal quality or sensor configuration. Last, the proposed Motion Tape sensing platform has not been tested during an actual boxing training event or match; when this happens, other hardware and software issues may arise. For example, hardware issues can include moisture and body temperature changes that affect Motion Tape’s electrical properties [37] or how the detachment and loss of a particular Motion Tape would affect algorithm performance. On the other hand, signal latency, or system responsiveness, has not been assessed and needs to be further investigated.

## 4. Conclusions

This study successfully demonstrated the potential of using machine learning algorithms to process Motion Tape wearable sensor data for assessing boxing performance, including punch detection and punch classification. By integrating a custom DAQ to synchronize and collect Motion Tape sensing streams from multiple body locations during a human participant study, the chosen punch detection algorithm and multivariate time series classification machine learning model were able to successfully detect punch events with an *F*_1_ score of 95% and an overall accuracy of 96.9%. These findings lay the groundwork for developing a pipeline to evaluate boxer performance using novel wearable sensor data that informs physical movements and muscle engagement. Overall, this work lays the groundwork for how this Motion Tape hardware–software platform could be utilized for other sports and physical training applications. For example, for golf, the methodology could be used to identify probable biomechanical causes of poor golf shots. Future work will include refining the punch detection algorithm to improve its detection accuracy, collecting more punch types, exploring other techniques for fatigue analysis and punch strength estimation, developing a wire-free Motion Tape sensing platform, and validating and polishing the approach for use in real-world training scenarios.

## Figures and Tables

**Table 1:** Summary of types of punches under different conditions.

Set Number	Punch Types	Number of Trials	Weight	Shadowboxing	Description
1	Jab	5	-	Yes	Jabs shadowboxing
2	Jab	5	5 lb	Yes	Jabs shadowboxing with 5 lb dumbbell
3	Jab	2	-	No	Jabs striking heavy bag
4	Lead hook	5	-	Yes	Lead hooks shadowboxing
5	Lead hook	5	5 lb	Yes	Lead hooks shadowboxing with 5 lb dumbbell
6	Lead hook	2	-	No	Lead hooks striking heavy bag

**Table 2:** Optimized hyperparameters used for machine learning models.

Models	Batch Size	Learning Rate	Dropout			Max Dilations	Number of Filters	Conv ks	Number of Layers
			Encoder	Fully Connected	Conv				
TST	39	1.386 × 10^−3^	1.084 × 10^−5^	3.815 × 10^−5^	-	-	-	-	4
MiniRocket	32	0.560	-	1.047 × 10^−5^	-	39	-	-	-
InceptionTime	31	1.072 × 10^−3^	-	1.125 × 10^−4^	5.544 × 10^−5^	-	25	28	-

**Table 3:** Punch detection results of each class.

Punch Types	${Acc}_{punch}$	$R^{D}$	$P^{D}$	$F_{1}^{D}$
Jabs	92.5%	92.5%	100%	96.1%
Jabs (5 lb)	89.6%	89.6%	100%	94.5%
Jabs (BOB)	97.6%	97.6%	100%	98.8%
Lead hooks	88.8%	100%	88.8%	94.1%
Lead hooks (5 lb)	99.0%	100%	99.0%	99.5%
Lead hooks (BOB)	70.4%	97.4%	71.7%	82.6%
Overall	90.5%	95.8%	94.3%	95.0%

**Table 4:** Punch classification results of each class.

Punch Types	TST	MiniRocket	InceptionTime
Jabs	100%	100%	91.7%
Jabs (5 lb)	100%	91.7%	91.7%
Jabs (BOB)	75.0%	75.0%	50.0%
Lead hooks	100%	92.3%	92.3%
Lead hooks (5 lb)	94.4%	94.4%	100%
Lead hooks (BOB)	100%	100%	100%
Overall	96.9%	93.8%	92.3%

## Data Availability

The data presented in this study are available upon request from the corresponding author. The data are not publicly available, owing to ethical concerns, as they were obtained in a clinical trial.

## Abbreviations

The following abbreviations are used in this manuscript:

SVM	Support Vector Machine
IMU	Inertial Measurement Unit
DAQ	Data Acquisition
GNS	Graphene Nanosheets
EC	Ethyl Cellulose
ETOH	200 Proof Ethyl Alcohol
K-Tape	Kinesiology Tape
MCU	Microcontroller Unit
ADC	Analog-To-Digital Converter
BLE	Bluetooth Low Energy
SLA	Stereolithography
MOCAP	Motion Capture
IR	Infrared
PCB	Printed Circuit Board
PC	Personal Computer
BOB	Body Opponent Bag
TST	Time Series Transformer
ML	Machine Learning
MiniRocket	Minimally Random Convolutional Kernel Transform
CMA-ES	Covariance Matrix Adaptation Evolution Strategies
PSO	Particle Swarm Optimization
Cobyla	Constrained Optimization By Linear Approximation
Fast-GA	Fast Genetic Algorithm
LLM	Large Language Model
NLP	Natural Language Processing
PPV	Proportion Of Positive Values
CNN	Convolutional Neural Network
